# NiO Grained-Flowers and Nanoparticles for Ethanol Sensing

**DOI:** 10.3390/ma13081880

**Published:** 2020-04-16

**Authors:** Marilena Carbone, Pietro Tagliatesta

**Affiliations:** Department of Chemical Science and Technologies, University of Rome Tor Vergata, Via della Ricerca Scientifica 1, 00133 Rome, Italy; Pietro.Tagliatesta@uniroma2.it

**Keywords:** NiO, multishaped nanoparticles, gas sensing

## Abstract

Grained-flower and nanoparticles NiO samples were synthesized with a straightforward, surfactant-free hydrothermal procedure, and probed with respect to ethanol gas-sensing. Both morphologies displayed excellent performances in terms of gas response vs. temperature and concentration and are very reproducible. The grained-flower, however, performed better than the nanoparticles NiO, probably due to the shorter travelling distance of the electrons and/or adsorbates during the detection process. Both sensors displayed high stability over three weeks. The grained-flower NiO sensor also has a good selectivity.

## 1. Introduction

Ethanol as a volatile organic compound (VOC) requires accurate and fast sensing since it is pivotal in monitoring chemical reactions, breath analysis, food control, and biomedical productions. Therefore, several types of sensors have been developed based on metal oxides, conducting polymers carbon nanostructured materials, and porous materials [1,2,3,4,5,6,7,8,9,10,11,12,13,14]. Among metal oxides, NiO is a p-type semiconductor with a wide band gap (3.0–4.0 eV) [15,16,17] and supercapacitor properties extensively applied in electrochemical devices, lithium ion batteries, and dye-sensitized photocathodes [18]. The efficiency of the applications is often morphology-related and different shapes of NiO have varied responses, modulated through the combination of porosity and surface area. When dealing with gas sensing [19], and in particular ethanol sensing by NiO-based devices, nanoblocks, nanorods, nanowires [20], nanosheets, nanobulks, nanospheres [21], flake-flowers [22], nanoplates assembled by nanosheet arrays samples, [23] and nanosheets with neck-connected networks [24] were probed among the others, with optimal operational temperature in the 200–350 °C range for concentrations of 50–500 ppm. The tuning of the synthesis parameters is often the key to modulate nanoparticles’ size and morphology and associated performances [25,26]. Shapes of the nanosized NiO samples are often varied *via* hydrothermal synthesis by changing parameters, such as the types of nickel salts, alkali, solvent, or the additional surfactant and ensuing calcination temperature [27,28,29]. In the perspective of widespread applications, the simpler the synthesis, the more suited for practical purposes. Employing surfactant-free and templating agent-free reactions has the advantage of avoiding pollutions from incomplete removal of the reaction-directing agents. Hydrothermal synthesis has the option of directing structure and the morphology of the products by fine tuning of the physical and chemical parameters, i.e., temperature, reagents concentration and reaction time. Here, we explore the ethanol gas-sensing properties of NiO synthesized using urea as alkali in an aqueous, surfactant-free hydrothermal synthesis. Urea has a shape-addressing role, in hydrothermal synthesis of the Ni(OH)_2_, a NiO precursor, which is then converted into NiO by calcination. By varying the ratio between Ni salt and urea, nanowires or nanoparticles can be obtained. The nanowire morphology is eventually retained upon calcination at 500 °C to afford the corresponding NiO [30]. Other types of NiO preparations by the use of urea include the combustion route [31] and the urea-melt assisted synthesis [32] among others. In the present paper, the hydrothermal synthesis of Ni(OH)_2_ with urea is followed by calcination at 400 °C and 600 °C. The NiO calcined at 400 °C, in particular, is characterized by nanoparticles of the order of 10 nm, arranged in a sort of grained-flowers, with sizeable nanoparticle interdistance for gas adsorption and desorption, and ensuing excellent performances in gas-sensing. The calcination at 600 °C causes a merging of the nanoparticles to an average 25-nm size, with a less organized superstructure, lower surface area, and correspondingly lesser performances in ethanol detection.

## 2. Materials and Methods

The NiO samples were prepared via hydrothermal synthesis using Ni(NO_3_)_2_ (Sigma-Aldrich, St. Louis, Missouri, USA 98.5% pure) and (NH_2_)_2_CO (Sigma-Aldrich, St. Louis, MO, USA 98.0% pure) followed by calcinations either at 400 °C or 600 °C. Typically, 50 mL of 0.9 mol L^−1^ Ni(NO_3_)_2_ were placed in a beaker and added with 50 mL of (NH_2_)_2_CO 1.8 mol L^−1^ dropwise, at room temperature under vigorous stirring (the aqueous solutions were prepared with distilled water). The pH was adjusted to 8.0 by using HNO_3_ (Sigma-Aldrich, St. Louis, MO, USA 90%). The suspension was, then, transferred into a 200-mL Teflon-lined stainless-steel autoclave, carefully sealed, and heated up at 180 °C for 24 h in a furnace. After a gentle cooling, the powder was repeatedly washed with deionized water and dried at 60 °C overnight, to yield a greenish Ni(OH)_2_ powder. The powder was subsequently placed in a tubular oven and heated up to the target temperature, at a rate of 10 °C/min. Calcinations were carried out for 3h in air atmosphere. The specific surface area and porosity were measured on Micrometrics Instrument Corporation ASAP 2020 (Micrometrics Instrument Corporation, Norcross, GA, USA) using N_2_ adsorption–desorption isotherms. Ni(OH)_2_ and NiO samples were characterized by X-ray diffraction (XRD), using an X’ pert pro X-ray diffractometer by Philips (Almelo, The Netherlands), operated with CuK-Alpha radiation. Scanning Electron micrographs (SEMs) of the oxides were collected with a Zeiss Auriga Field Emission-Scanning Electron Microscope (Jena, Germany) instrument operating at 7 kV. 

Gas sensing properties were probed with in-house extension of the MQ-3 Your Cee, gas sensitivity instrument in a static system controlled by a central computer. The NiO powders were grinded in an agate mortar, mingled with deionized water in a ratio of 5:1 until a paste was formed, and then coated onto the ceramic support, connected to 4 Pt wires through 2 Au rings. The sensors were then dried at 270 °C for 3 h in air, for water to evaporate and ensure stability. The thickness of the coating was 200 μm. The sensor was placed in a chamber of 1 L of volume, with a heating system and a temperature control. Synthetic dry air (Sigma-Aldrich, St. Louis, MO, USA), was used both as reference and as diluting gas. Ethanol (Sigma-Aldrich, St. Louis, MO, USA 99.8% pure) saturated vapor was injected in the test chamber with a microsyringe. The resistance was then measured with a signal-to-noise ratio of about 4% at the minimum level and about 2% at the maximum level.

## 3. Results and Discussion

**Figure 1:**
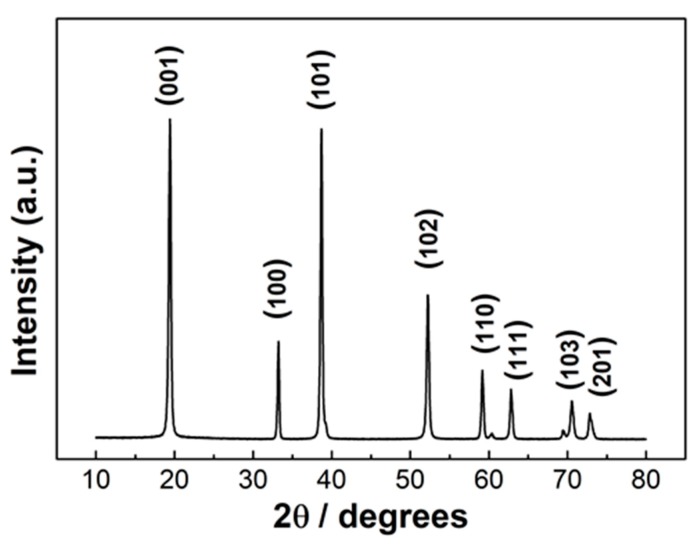
XRD pattern of β-Ni(OH)_2_, obtained by hydrothermal synthesis with urea.

The X-diffraction pattern of the powder obtained after hydrothermal synthesis and prior to the calcination is reported in Figure 1. The diffraction peaks at 19.4°, 33.3°, 38.7°, 52.2°, 59.2°, 62.9°, 70.4°, 73.0° were attributed to the reflections of the planes (001), (100), (101), (102), (110), (111), (103), (201) respectively of β-Ni(OH)_2_ [33]. 

**Figure 2:**
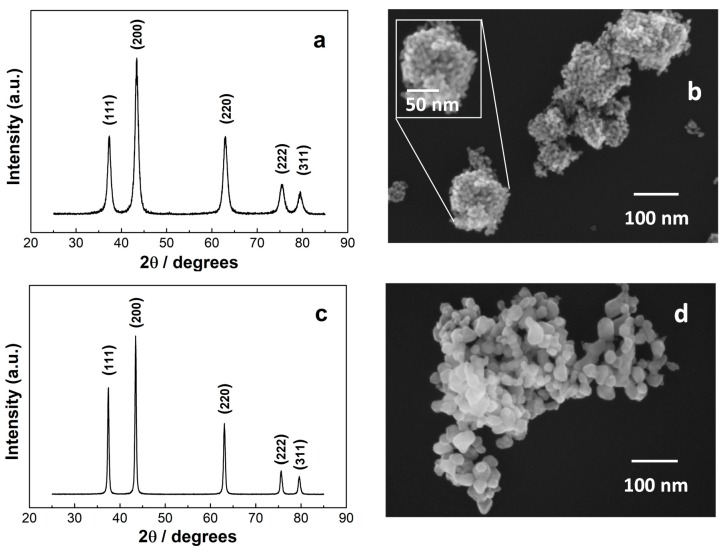
(**a**) XRD and (**b**) SEM image of grained-flowers NiO (**c**) XRD and (**d**) SEM images of the nanoparticles NiO. The inset in panel (**b**) is the enlargement of one of the grained-flowers.

The characterizations of the NiO samples obtained upon calcinations are reported in Figure 2. The X-ray diffraction pattern and the SEM image of the sample calcined at 400 °C are reported in panels a and b, respectively. The X-ray diffraction pattern and SEM image of the sample synthesized at 600 °C are sketched in panels c and d. Both samples display 5 diffraction peaks in the 35–85 2θ range, at 37.4°, 43.4°, 63.0°, 75.5°, 79.6°, which were assigned to the reflections of the planes (111), (200), (220), (222), and (311), respectively [34]. No other peaks of other phases or impurities were detected. The assigned reflections correspond to a face centered cubic crystalline structure, quite often achieved upon calcination of a hydrothermally synthesized Ni-containing precursor and common to all the NiO-based sensors of comparison in this paper [15,17,18,19,20,21,22,23,24,27,28,29,30,31,32]. The full width at half maximum was used to estimate the average size, through the Scherrer formula, D = K λ / β cos (θ), where K is a constant (ca. 0.9), λ is the X-ray wavelength used to collect the XRD patterns (i.e., 1.5418 Å), θ is the Bragg angle, β is the pure diffraction broadening of a peak at half-height due to the crystallite dimensions. The estimated average size is 10 nm for the NiO sample calcined at 400 °C and 25 nm for that obtained at 600 °C. The morphology of the samples is quite different in the two cases, as can be seen from the SEM images. At lower temperature, the 10-nm diameter nanoparticles can be observed. The size distribution is quite regular and statistics over 144 nanoparticles yielded an average size of 10 ± 1 nm. The 3D arrangement of the nanoparticles produces superstructures to some extent similar to flowers, which we labelled as grained-flowers. The calcination at higher temperatures causes a sintering of the nanoparticles, with a consequent enlarging of the surface over the volume and creation of nanosized particles. The average particles size in this case is 25 ± 15 nm. The variation of the grey scale when moving from one nanoparticle to the other is compatible with a height smaller than the particle diameter, hinting at a “slice” shape.

The surface area of the NiO samples is reported in Table 1 and it is higher for the grained-flower as compared to the nanoparticles, as could be expected from smaller particles, with a higher surface-to-volume ratio.

**Figure 3:**
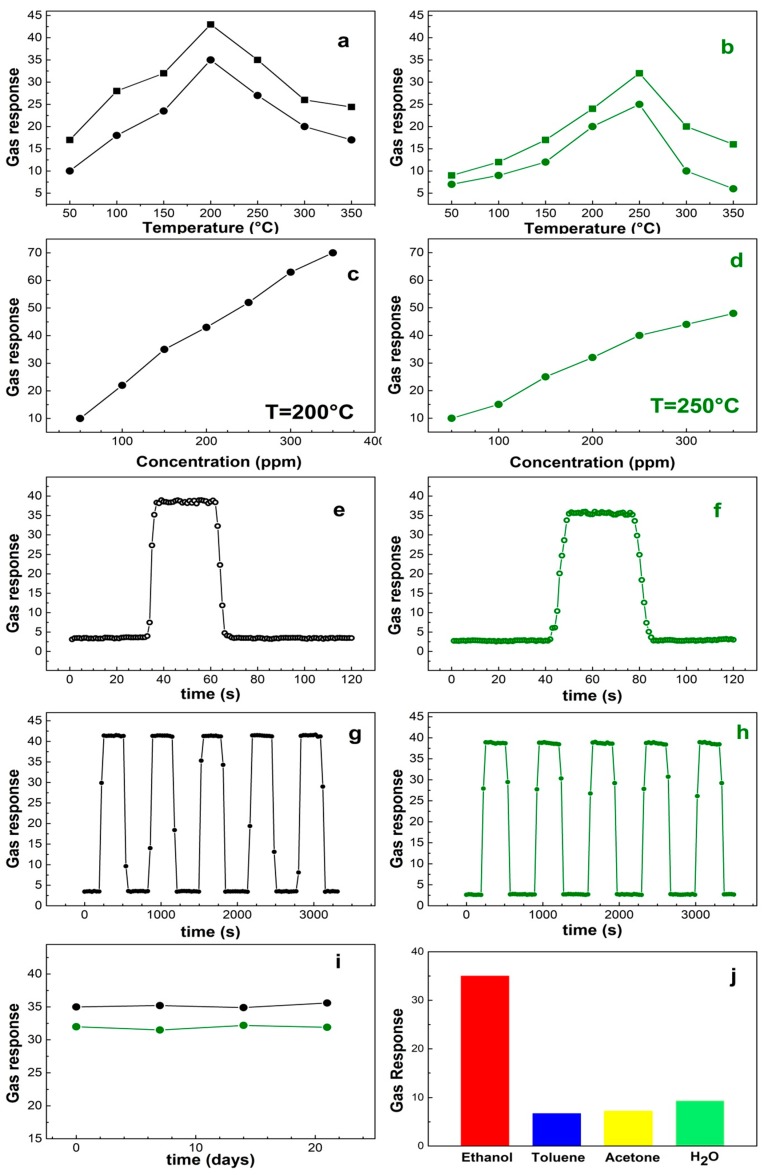
Plots of the gas responses. (**a–h**): the left panels correspond to grained-flower NiO, the right ones to the NiO nanoparticles. (**a**,**b**) are the gas response vs. temperature of sensors exposed to 150-ppm (solid dots) and 200-ppm (solid squares) ethanol, respectively. The gas responses vs. concentration are reported at 200 °C for the grained-flower (**c**) and 250 °C for the nanoparticles (**d**). (**e**,**f**) are response/recovery time; (**g**, **h**) repetitivety over time. Panel (**i**) is the response of the grained-flower sensor (black line) at 200 °C to 150-ppm ethanol and nanoparticles sensor (green line) to 200-ppm ethanol at 250 °C, over time (3 weeks) and (**j**) the response of the grained-flower sensor at 200 °C to 150-ppm ethanol, toluene, acetone, and water.

The gas sensing properties of NiO grained-flowers and nanoparticles were probed by monitoring the response as ratio between air and gas resistance (Rg/Ra, Rg = gas resistance, Ra = air resistance), as a function of several parameters. In particular, the response vs. temperature and concentration were tested along with response/recovery time, reproducibility, and stability tests. The corresponding plots are comparatively reported in Figure 3a–h, where the left panels all correspond to the grained-flower NiO performances, the right ones to the nanoparticles NiO. Figure 3i is the stability plot of both morphologies and Figure 3j plots the selectivity of the grained-flower NiO sensor. The overall response of two morphologies of NiO under investigation is excellent, with low operational temperature and linear response vs. gas concentration in the 100–700 ppm range. In detail, the grained-flower display an optimal temperature of 200 °C and 250 °C, respectively, i.e., below or equal to the temperatures reported for ethanol sensing by other NiO morphologies [13], though with a better Ra/Rg ratio.

In Figure 3a,b, the response vs. temperature is reported for 2 concentrations, i.e., 150 ppm and 200 ppm. The response and recovery times (the time taken to achieve 90% of the final change [35,36]) are nearly 3 s/6 s for the grained-flowers, 4 s/8 s for the nanoparticles. We estimated the limit of detection (LOD) as:

(1) $$\mathrm{LOD}=3\frac{rms_{noise}}{slope}$$

where *rms_noise_* and *slope* are the root-mean-square error of the baseline and the slope value of the linear curve [37]. Accordingly, the LODs of the NiO grained-flowers and nanoparticles sensors are 2.6 ppm and 2.4 ppm, when operated at 200 °C and 250 °C respectively. The repetitively is excellent for both samples as can be seen in Figure 3g,h. The stability of the sensors has been probed by testing the response to 150 ppm ethanol at 200 °C (grained-flower) and 200 ppm ethanol at 250 °C (nanoparticles) in a time span of 3 weeks. The outcome is reported in Figure 3i and denotes a remarkable stability. Selectivity to a target gas is always an important factor for a gas sensor. Figure 3j shows the measured selectivity of the grained-flower NiO sensor to 150 ppm of toluene, acetone and water, when operated at 200 °C. The response to ethanol is 35%, whereas the response to the other gases is 6.7%, 7.2%, and 9.3%, for toluene, acetone, and water, respectively. The latter data are of particular interest, they give indications that the sensor can be used in humid environments. The root of the good performances of these two morphologies can be related to the sensing mechanism, which is hypothesized to be based on adsorption properties at the surface, ensued by redox reactions. The air oxygen is adsorbed at the NiO surface and reduced by electrons of the conduction band to $O_{x}^{y-}$ with consequent holes accumulation at the surface. Incoming ethanol is then fully oxidized to CO_2_ via $O_{x}^{y-}$, thus back releasing electrons to NiO [20,21,23,24]. In this scenario, surface area, porosity, and band gaps play a role in determining the efficiency in gas sensing, since they are related to the capability of the material to capture the molecules and act as electron vehicle in the redox reactions. Grained-flowers and nanoparticles exert these properties in different ways. From one side, grained flowers have a larger surface area than nanoparticles, a factor that enhances the gas response. On the other side, nanoparticles have smaller band gaps as compared to bulk materials, the smaller the particle, the smaller the band [20] down to a limit value where quantum confining becomes significant [38]. Furthermore, nanoparticle boundaries can be both sources of internal holes and absorbers of the traps. Grained-flowers, being aggregations of 10-nm nanoparticles are bound to have a large amount of electrons available for surface reactions. The corrugated surface and firm interparticle distance also provide a steady surface for gas adsorption. The NiO nanoparticles have charges/holes accumulations at the short dimension, where more defects and traps accumulate and has an extended surface area in the long direction. This combination also allows an efficient gas detection, though not at the levels of the grained-flowers NiO. This is partly due to the location in different areas of the charge transfer processes and reactant adsorptions, so that electron mobility through the NiO particle and/or surface adsorbates mobility are required for the detection to occur. This also results in delayed response/recovery of the nanoparticles with respect to the grained-flowers. In order to get an overview of the ethanol sensing properties in the panorama of varied NiO morphologies, a brief summary is reported in Table 2 including literature data vs. the current paper. The comparison reveals that NiO grained-flowers and nanoparticles have better or at least comparable performances with respect to the other NiO morphologies.

## 4. Conclusions

In summary, we have probed ethanol gas sensing devices built up with NiO nanoparticles arranged in two unprecedentedly explored morphologies: grained-flowers and nanoparticles. The synthesis of the NiO is a straightforward, surfactant-free one, with ensuing calcination at different temperatures. The gas sensing response vs. temperature and concentration are excellent, with the grained-flower NiO performing better, probably due to a shorter travelling distance of the electrons in the solid, during the sensing process. Stability over three weeks of both sensors and selectivity of the grained-flower NiO sensor are also good.

## Figures and Tables

**Table 1:** Surface area of the synthesized NiO samples.

Sample	Surface Area (m^2^g^−1^)
**Grained-flowers**	240.3
**Nanoparticles**	170.1

**Table 2:** Comparison of the performances of different NiO based ethanol sensors. UT=Ultrathin.

Sample	Ethanol (ppm)	Operating Temperature	Rg/Ra	Response/Recovery Time (s)	Ref.
**Grained-flower**	150	200	35	3/6	This work
**Nanoparticles**	200	250	32	4/8	This work
**UT-Nanosheet**	200	200	3.1	N/A	[24]
**Flake-flower**	400	300	32	4/8	[23]
**Nanoblocks**	500	300	1.1	5/5	[20]
**Nanorods**	500	300	1.4	4/5	[20]
**Nanowires**	500	300	3.4	5/6	[20]
**Nanosheet**	50	350	1.8	6/9	[21]
**Nanobulks**	50	350	1.5	5/7	[21]
**Nanospheres**	50	350	2.5	8/5	[21]
